# Anesthesia for non-traditional consciousness

**DOI:** 10.3389/fnhum.2023.1146242

**Published:** 2023-05-09

**Authors:** Ben Deverett

**Affiliations:** Department of Anesthesiology, Perioperative and Pain Medicine, Stanford University, Stanford, CA, United States

**Keywords:** anesthesia, consciousness, artificial intelligence, brain machine integration, ethics, animal consciousness, extraterrestrial life

## Introduction

The idea of conscious experience occurring in non-human systems has received attention across philosophy (Shevlin, 2021), biology (Trewavas and Baluška, 2011), and computational (Tononi et al., 2016; Dehaene et al., 2017) research. There is increasing recognition that we will likely encounter these new forms of consciousness; for example, in artificially intelligent (AI) systems (Reggia, 2013; Dehaene et al., 2017), brain-computer interface (BCI) technologies (Bernal et al., 2021), or interaction with extraterrestrial life (Merali, 2015; Schneider, 2016). While progress has been made in studying the basis of animal consciousness, there is limited consensus on how we may characterize and engage with consciousness that is not human, animal, or even biological in origin. These non-traditional consciousnesses will present unique challenges because without relatable behaviors to help us infer their internal experiences, we are at risk of under- or over-utilizing our ability to interpret and compassionately influence them. Tackling this challenge will be a multidisciplinary endeavor. Anesthesiology, a profession dedicated to safely and effectively altering conscious states in the service of comfort, brings a unique perspective to this frontier (Mashour, 2006; Bonhomme et al., 2019).

This discussion is valuable for many reasons. It enables us to explore how we might recognize awareness and discomfort in the absence of familiar biology. It prompts us to reflect upon the reasons we manipulate consciousness, and to consider how we may abstract the concept of the anesthetic state to beings of entirely different composition. It also presents impactful ethical considerations: manipulation of consciousness can be a powerful tool to alleviate suffering, but it is accompanied by a unique forfeit of autonomy. What principles can we devise to guide our interactions with non-traditional consciousness, and how can existing practices from relevant professions like anesthesiology inform these decisions?

Given the complexity of consciousness, it is pertinent to maintain an open discussion on this topic, acknowledging we are bound to have more questions than answers at this stage. This article draws on lessons from consciousness science and anesthesiology as an introductory discussion into interaction with non-traditional consciousness.

## Defining consciousness

Consciousness has historically been a controversial topic in part due to challenges in precisely defining it (Seth and Bayne, 2022). Here the term refers to any ongoing subjective experience occurring within an entity (Nagel, 1974), with a focus on that which can conceivably be altered in a deliberate fashion. The existing literature on consciousness has helped clarify defining features of conscious states, such as the distinction between consciousness *level* (how aware an entity is) vs. *contents* (which experiences are occurring), and between *functional* aspects of experience (e.g., how one experience takes conscious priority over another) vs. *phenomenal* ones (e.g., the experiential difference between seeing two different colors) (Michel et al., 2019; Seth and Bayne, 2022). This research is largely neurobiologically oriented, focusing on the basis of subjective experience in humans (Boly et al., 2013; Mashour and Alkire, 2013; Hohwy and Seth, 2020; Mashour et al., 2020) and other organisms (Trewavas and Baluška, 2011; Calvo et al., 2017; Baluška and Reber, 2019). Other work considers more generalized definitions, such as measures of information processing that could apply to living and non-living systems (Tononi et al., 2016). Theories like panpsychism argue that consciousness is a quality inherent to matter, though to date these offer limited testable predictions for scientific study (Goff, 2019; Matloff, 2020; Seth, 2021). Notwithstanding subtleties in definition, it stands that we will likely encounter entities which convincingly demonstrate that they have experience, motivating us to apply our moral standards to them. Given the anesthesiologist's focus on manipulating conscious states in the service of comfort, it is those scenarios that take focus here.

## Sources of consciousness

At present there is a short list of entities—just specific animals—whose consciousness we intentionally and directly intervene upon, via medical and veterinary anesthesiology. This animal consciousness has been studied extensively in neuroscience. Brain imaging and perturbation studies suggest that conscious experience arises from network-level activity of neurons arranged in precise connectivity structures; whether the relevant networks are primarily cortical/subcortical or frontally/posteriorly located is an ongoing area of investigation (Seth and Bayne, 2022). This has raised the question of exactly when organisms evolved to meet criteria for consciousness, and accordingly there has been work on the biological evolution of consciousness as well (Mashour and Alkire, 2013; Kelz and Mashour, 2019).

However, a nervous system like ours is not conceptually necessary for consciousness. For example, there is active debate over whether plants exhibit consciousness (Calvo et al., 2017; Draguhn et al., 2021), and this demonstrates the challenges in proving the presence of subjective experience when its physical substrate differs from that of our own. For this reason it is helpful to consider characterizations of consciousness that do not require biology: one example, Integrated Information Theory (Tononi et al., 2016) (IIT), proposes that consciousness arises when a critical degree of information integration occurs; this threshold is presumably reached in the brain but may also be reached anywhere information can be integrated. Some critics view IIT as insufficiently specific, citing simple systems that meet its criteria without convincingly demonstrating consciousness (Cerullo, 2015). Notwithstanding its limitations, theories like this demonstrate how we may formally approach substrate-independent notions of consciousness without the need for specific biological underpinnings.

In this view, numerous biological and non-biological entities could conceivably exhibit consciousness. For discussion purposes we can categorize these based upon features that may help us interact with them: (i) Communication without biology [C^+^B^−^, e.g., AI agents (Krauss and Maier, 2020) and certain BCIs (Goertzel and Ikle, 2012)]; (ii) Biology/organic substrate without communication [C^−^B^+^, e.g., plants, (Calvo et al., 2017) other biological organisms, and likely alien lifeforms (Cockell, 2016)]; (iii) Neither communication nor biology (C^−^B^−^, e.g., complex integrated non-biological systems).

Establishing specific criteria by which to identify these foreign consciousnesses is a deeply non-trivial task sometimes called the Measurement Problem (Michel, 2019; Browning et al., 2020). An example of this challenge is with modern AI language models (Brown et al., 2020), where communication passes the Turing Test (Turing, 1950) but consciousness is not widely thought to be present. This article does not propose criteria to solve the Measurement Problem but rather focuses on how we might engage with entities when we suspect these criteria are met. Perhaps in the future, augmentation of human consciousness via BCIs can help bridge our subjective experience with other types of consciousness and resolve uncertainties regarding non-traditional consciousness. For now, while extrapolation from human consciousness may be a flawed approach to characterizing other consciousness (Browning et al., 2020), employing knowledge of our own conscious experience is a practical starting point.

## Gauging consciousness

Anesthesiologists are trained to recognize signs of consciousness using metrics gleaned from behavior and physiological measurements. Specifically, we are tasked with inferring others' internal experiences and maintaining their comfort both before and after inhibiting means of communication. To do so, we monitor various proxies as windows into experience, which can broadly be categorized into groups (Table 1A): (1) explicit communications with the subject, (2) measurement of processes putatively responsible for consciousness itself, and (3) measurement of physiologic processes occurring as a result of specific conscious experiences.

**Table 1:** Principles from anesthesiology as a generalized framework for interaction with consciousness.

**A) Approaches to inferring level and contents of consciousness**	**Examples in modern anesthesiology practice**
Explicit communication and instruction-following	Clinical interview; scoring systems such as the Glasgow Coma Scale
Measurement of processes underlying consciousness	EEG-based depth-of-anesthesia monitors
Assessing downstream consequences of internal experiences	Inference of arousal and pain levels using physiologic variables (e.g., heart rate, blood pressure, and respiration)
**B) Criteria for influencing consciousness**	**Examples in modern anesthesiology practice**
Recognition or expectation of significant physical pain or discomfort	Medication to induce unconsciousness and amnesia for the duration of a surgery, or for procedures in ICU
Protection from intolerably disturbing experiences; prevention of damaging memory formation	Sedation in surgeries where pain is already eliminated by regional/local anesthesia approaches; sedation for non-invasive imaging studies in vulnerable patients
Mitigation of active risk of harm to self or others	Temporary sedation for states of aggravated agitation
**C) Properties of consciousness-influencing tools**	**Examples in modern anesthesiology practice**
Complete reversibility	Anesthetic medications clear from the body without residual sedation; explicit reversal agents also exist for some medications
Titratable degree of consciousness disruption	Extent of sedation is controlled by medication dosage, which is monotonically related to effect
Continually measurable consequences	Continuous anesthetic monitors are mandatory to assess and adjust the effects of anesthesia

As an example of these principles, consider a typical perioperative workflow. We first perform a preoperative assessment, estimating the patient's internal experience using proxy #1: verbal and non-verbal communication during clinical interview and examination. We next deliver medications that sedate and paralyze the patient, eliminating their ability to overtly communicate, at which point we must employ other proxies. In many instances, we directly evaluate brain activity responsible for conscious experience using electroencephalographic (EEG)-based monitoring, which aggregates high-dimensional population neuronal activity into low-dimensional indices of depth of anesthesia (Fahy and Chau, 2018). This example of proxy #2 helps assess the degree of consciousness disruption [not necessarily equatable with level of consciousness *per se* (Hudson et al., 2014)], but is often inadequate for evaluating contents of experience, such as pain. Unlike anesthetic depth monitors, reliable direct measurement of pain processing is not yet available (Ledowski, 2019). However, pain and brief periods of increased awareness can be detected by the stress response they trigger, measurable as elevated heart rate, respiratory rate, and blood pressure, as well as changes in the amount of medication required to stabilize those variables. These measurements are examples of proxy #3: here we infer conscious experiences not based upon direct communication or neuronal measurements, but rather by observing their downstream consequences.

This general framework can be extrapolated to non-traditional consciousness, where our ability to assay experience is limited by fundamental differences in the mechanisms and substrates of the consciousness itself. The utility of the above measurement proxies depends upon the type of non-traditional consciousness in question. Proxy #1 (communication) is the most straightforward but is available only for C^+^B^−^. To use proxy #2 (measuring mechanistic processes), if we consider the hypothesis that integrated information underlies consciousness, we might engineer a monitor analogous to the aforementioned anesthetic depth monitor to measure informational integration and convert it into an interpretable metric correlated with consciousness. When applied to entities who share features of our biology (C^−^B^+^), we may apply tools from neuroscience or molecular biology to measure cellular processes that are likely to underlie their experiences. Without shared biology (C^+^B^−^ and C^−^B^−^), the challenge is to identify specific processes to measure [e.g., activations of units in a neural network (Deverett et al., 2019; Bau et al., 2020), or states of transistors in electrical circuit-based assemblies]. For proxy #3 (measuring consequences of experience), the range of possible measurable behaviors is vast. As one pertinent example, to monitor for pain or distress we might search for processes analogous to a stress response: C^−^B^+^ offers the advantage that stress has recognizable features shared across biology (Kültz, 2020), whereas outside biology (C^−^B^−^ and C^+^B^−^) we might identify an increase in energy expenditure beyond a typical distribution, or signs of deviation from a baseline state that is refractory to attempts at correction. In all cases, we should monitor for evidence of memory formation, which predisposes to the possibility of lasting trauma. We can also draw on the sciences of sleep (Windt et al., 2016) and dreaming (Cascella et al., 2017; Chow et al., 2022), meditation (Raffone and Srinivasan, 2010), and psychedelics (Millière et al., 2018; Williams et al., 2018; Yaden et al., 2021), all of which shed light on altered states of consciousness.

## Influencing consciousness

Once consciousness has been recognized and characterized, we must consider our influence upon it. The decision to influence consciousness is profoundly impactful and complex, involving considerations of autonomy, competence, and capacity (van Norman and Rosenbaum, 2020). Defining this boundary will be challenging with new forms of consciousness, especially when explicit communication is unfeasible. As an example of the potential complexity, consider a conscious AI that undergoes a maintenance update of its hardware. If we disable a subset of its modules while failing to proactively pause its conscious experience, we run the risk that this anomalous experiential data point could not only cause a disturbing subjective experience, but could train it to form maladaptive and irrational behaviors. We thus need formal criteria to justify the manipulation of consciousness and guard against inappropriate uses—intentional or unintentional—of our abilities to influence experience.

Intuitions that have developed through the practice of anesthesiology may serve as a framework. Table 1B outlines criteria under which we often choose to administer anesthesia and which may generalize to new, unfamiliar circumstances: to eliminate pain and discomfort, to prevent disturbing experiences and memory formation from them, and to mitigate risk of harm to self and others. If we deem it appropriate to influence consciousness, we must then consider how to effect that intervention. In humans, this amounts to preparing an anesthetic plan by selecting from the array of anesthetic agents and critically evaluating their benefits and harms on a case-by-case basis. When devising such a plan to apply to a novel conscious entity, what approaches might we consider? In the simplest case, we can examine where our existing anesthetics might translate to the entity in question; for example, volatile anesthetic agents have surprisingly broad efficacy ranging essentially the span of life including animals, microorganisms, and plants (Kelz and Mashour, 2019), so they may be effective in select circumstances. However, the span of beings whose consciousness arises from organic material comparable to ours is likely a small fraction of the full range.

In considering how we might influence the consciousness of an entirely foreign system, we begin to contemplate a broader definition of anesthesia. As has been proposed, one may define the anesthetic state as the reversible interruption of informational exchange through a system of connected parts (Kelz and Mashour, 2019). Ultimately the mechanics of this informational disruption are modality-specific, but we can nevertheless prepare by anticipating which specific goals we aim to achieve, and by ensuring we develop anesthetic capabilities that deliver perturbations aligned with our values. These can again be extracted from the established practice of anesthesia: Table 1C identifies key examples of such properties: reversibility of interventions, titratability of their effects, and continually measurable consequences. Prior to inducing anesthesia, it is standard practice to meticulously prepare the equipment necessary to troubleshoot our disruption of consciousness and its complex physiologic sequelae. We then navigate unanticipated challenges before, during, and after our manipulation of consciousness, including prolonged effects such as delayed emergence (Misal et al., 2016; Cascella et al., 2020) and post-operative cognitive dysfunction (Berger et al., 2015). For non-traditional consciousness, we should maintain similar strict standards: appropriate monitors to ensure our degree of consciousness disruption is adequate and stable, and infrastructure to maintain, measure, titrate, reverse, and troubleshoot complications. These principles have crucial implications not only for the wellbeing of the subject, but also medicolegally for the practitioner, as is true in modern anesthesiology practice (Semo, 2014; Petrucci et al., 2021; Lee et al., 2022).

## Conclusion

Interaction with non-traditional forms of consciousness is likely to be part of humanity's future. If and when it materializes, there are likely to be disagreements on its veracity and importance, and on how to engage, which makes preparation all the more important. Anesthesiology has a unique perspective and role to play in informing our approach to this development. Drawing from clinical practices as in the examples above, we can begin to establish a framework for action. With ongoing research into conscious AI, brain-machine interfacing, non-human animal experience, and searches for extraterrestrial life, this frontier may become relevant sooner than we expect—and as is always true in anesthesiology, we ought to have a plan.

## Author contributions

The author confirms being the sole contributor of this work and has approved it for publication.
